# Evolution of Microsatellite Loci of Tropical and Temperate *Anguilla* Eels

**DOI:** 10.3390/ijms13044281

**Published:** 2012-04-02

**Authors:** Mei-Chen Tseng

**Affiliations:** Department of Aquaculture, National Pingtung University of Science & Technology, Pingtung 912, Taiwan; E-Mail: mctseng@mail.npust.edu.tw; Tel.: +886-8-7703202 (ext. 6227); Fax: +886-8-7740401

**Keywords:** flanking sequence, major region, polymorphic pattern

## Abstract

*Anguilla* eels are divided into temperate and tropical eels, based on their major distributions. The present study collected two temperate eels, *Anguilla japonica* and *Anguilla anguilla*, and two tropical eels, *Anguilla marmorata* and *Anguilla bicolor pacifica*, to examine two questions: do temperate and tropical *Anguilla* eels have different genetic polymorphic patterns?; and do temperate *Anguilla japonica* and *Anguilla anguilla* have a closer relationship to each other than to tropical eels? In total, 274 sequences were cloned and sequenced from six conserved microsatellite loci to examine polymorphic patterns of these four catadromous eels. Different mutational events, including substitutions, and repeat-unit deletions and insertions, appeared in major regions, while different point mutations were observed in flanking regions. The results implied that parallel patterns of microsatellite sequences occurred within both tropical and temperate freshwater eels. Consensus flanking sequences of six homologous loci from each of the four species were constructed. Genetic distances ranged from 0.044 (*Anguilla bicolor pacifica vs. Anguilla marmorata*) to 0.061 (*Anguilla marmorata vs. Anguilla anguilla*). The tree topology suggests the hypothesis of *Anguilla japonica* and *Anguilla anguilla* being a sister group must be rejected.

## 1. Introduction

**Figure 1:**
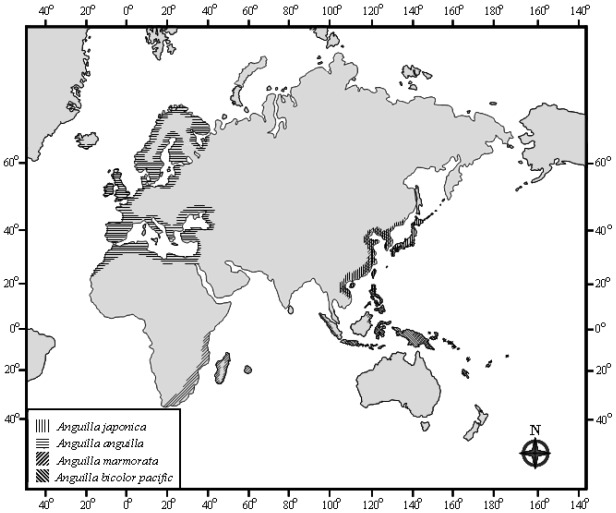
Distributions of four *Anguilla* species.

Freshwater eels of the genus *Anguilla* consist of 16 species and three subspecies [1] that are globally distributed in temperate, tropical, and subtropical areas. They all have a catadromous life-history strategy, spawning in remote tropical seas with larvae being transported back by currents to their nursery grounds in freshwater or estuarine areas. In general, they are divided into temperate and tropical eels, based on their major distributions (Figure 1) and ecological properties. For example, the temperate Japanese eel *Anguilla japonica* is extensively found in the northwestern Pacific Ocean. It spawns in summer, with juvenile recruits being transported back to the coasts of northeastern Asian countries in winter. The temperate European eel (*Anguilla anguilla*) is distributed in North Africa and Europe. It has a longer larval period than other *Anguilla* eels [2]. The tropical *Anguilla marmorata* is very common in the Indo-Pacific Ocean with a more-extensive distribution than temperate freshwater eels [3,4]. Adults of this species attain a greater maximum size than most temperate species [5]. Unlike the Japanese eel, *A. marmorata* spawns year-round, based on hatching dates of elvers inferred from otoliths [6]. *Anguilla bicolor pacifica* is a tropical short-finned eel, which occurs in natural waters of the Indo-Pacific area. However, little information on the biology of *A. bicolor pacifica* has been published elsewhere.

Several evolutionary hypotheses of freshwater eels were examined by morphological characterizations [3] and mitochondrial (mt) DNA [7–10]. *Anguilla* freshwater eels were divided into four groups, based upon color, body proportions, dentition, and meristic characters [3]. Temperate *A. japonica* and *A. anguilla* have more-similar morphological characters and were classified in the same group. In addition, previously published molecular systematics confirmed that the tropical *A. bicolor pacifica* and *A. marmorata* belong to an Indo-Pacific species lineage [9,10]. The mitochondrial molecular phylogeny, on the other hand, implied that *A. japonica* and *A. marmorata* belong to a Pacific group with a close relationship [8]. However, the two above-mentioned ideas are mutually incompatible. Whether *A. anguilla* and *A. japonica* are sister species is still open to question.

In recent years, scientists have begun to pay more attention to the advantages of using microsatellites as a way of studying evolution within species and between closely related species. Because of high polymorphism in the number of repeats, microsatellites are widely used as markers for studying genetic mapping, population structures, kinship, evolutionary genetics, and genetic diseases [11–16]. In addition to those advantages, some loci can also be well resolved to analyze high-level systematics by their flanking sequences. Asahida *et al*. successfully used flanking regions of microsatellite loci to distinguish rockfish species and study their phylogeny [17]. Zardoya *et al.* studied the phylogeny of the principal lineages of cichlid fishes based on DNA sequences of the flanking region of a microsatellite locus [18].

Microsatellites are the most rapidly evolving DNA sequences, with large mutation rates of 10^−5^–10^−2^ per generation [19,20]. Mutations at microsatellite loci generally involve a change in the repeat number [21]. Theoretical mutation models include the stepwise mutation model (SMM), infinite allele model (IAM), two-phase model (TPM), and K-allele model (KAM) [22–25]. The most likely mechanism for length variation is a mutation in the repeat number due to slippage during replication [20,26,27]. This slippage more frequently appears in microsatellites with greater numbers of tandem repeats [28–30]. In addition to the repeat number, mutations can also occur in the flanking region of microsatellites. However, efforts to determine the processes of microsatellite mutations have only recently begun in earnest. Therefore, sequencing alleles, both within and between species, is necessary, and may produce more details about microsatellite variations.

The present study attempted to explore the phylogeny of freshwater eels by applying nuclear markers. Two issues were examined: (1) a methodological one, looking at patterns of microsatellite sequences; and (2) a phylogenetic one, resolving the evolutionary relationship between eel species (*A. japonica* and *A. anguilla*). We examined 274 allelic sequences from six microsatellite loci to infer the mutation mechanisms of these loci among four *Anguilla* eels and explored the interspecific genealogy using consensus sequences of the flanking regions.

## 2. Results and Discussion

### 2.1. Microsatellite Variation

**Figure 2:**
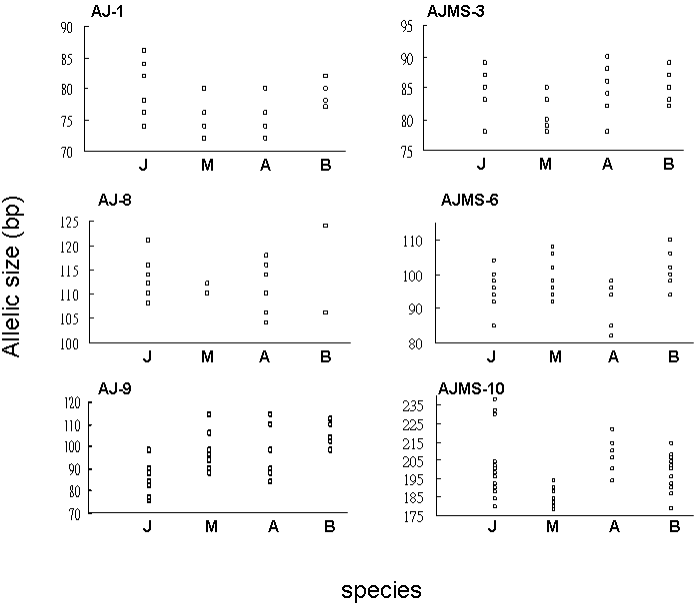
Allelic size distributions at six microsatellite loci, AJ-1, AJ-8, AJ-9, AJMS-3, AJMS-6, and AJMS-10, from four *Anguilla* species (J, *Anguilla japonica*; M, *Anguilla marmorata*; A, *Anguilla anguilla*; and B, *Anguilla bicolor pacifica*).

Mutations in microsatellite loci arise due to changes in repeat numbers and nucleotide substitutions. In total, 274 different colonies were successfully sequenced from the above six microsatellite loci of four *Anguilla* eels. Ranges of allelic sizes at the six loci from four *Anguilla* eels (Figure 2) indicated overlap across these species. Patterns of repeat regions at the six loci are listed in Table 1. The AJ-1 locus from 49 colonies was sequenced, with repeat numbers of five to 13 in the major region. An interrupted sequence from one individual was observed in *A. marmorata*. TG repeat numbers at the AJ-8 locus ranged 12–22. A substitution of TG/TA was only found in *A. anguilla*. The AJ-9 locus from 40 colonies was examined from the four *Anguilla* species. Interrupted nucleotide mutations, including TG/AG, TG/TA, and TG/CG, appeared in three of these freshwater eels, but not in *A. marmorata*. Allelic size variations were fully displayed by differences in numbers of repeat units. The repeat number of the dinucleotide, TG, at AJMS-3, ranged from seven to 11. Two interrupted sequences were only found in *A. anguilla*. Perfectly repeated sequences of the AJMS-6 locus were present in three species, but not in *A. bicolor pacifica*. Perfect GA repeat numbers of eight to 39 occurred at the AJMS-10 locus in the four species. Substitutions consisting of three mutations of GA/GG, GA/CA, and GA/AT in major regions only occurred in *A. bicolor pacifica* and *A. anguilla*. After sequencing, we observed that several types of mutations appeared in homologously-sized alleles with the same nucleotide number of the AJ-9 and AJMS-6 loci from *A. bicolor pacifica* specimens. Some alleles at the AJ-1 locus of *A. anguilla* also showed an identical size to those of other alleles, which resulted from the occurrence of one dinucleotide deletion in the flanking region.

Consequently, compositions of the major regions at the six microsatellite loci of *Anguilla* eels were generally categorized into two types: perfect- and interrupted-repeat sequences. No single-nucleotide indel was found in the major region of any of the six microsatellites from the four studied eel species. As to genetic variations in major regions of the six loci, no specific mutants appeared in the two temperate or tropical freshwater eels.

### 2.2. Flanking Region Analysis

**Figure 3:**
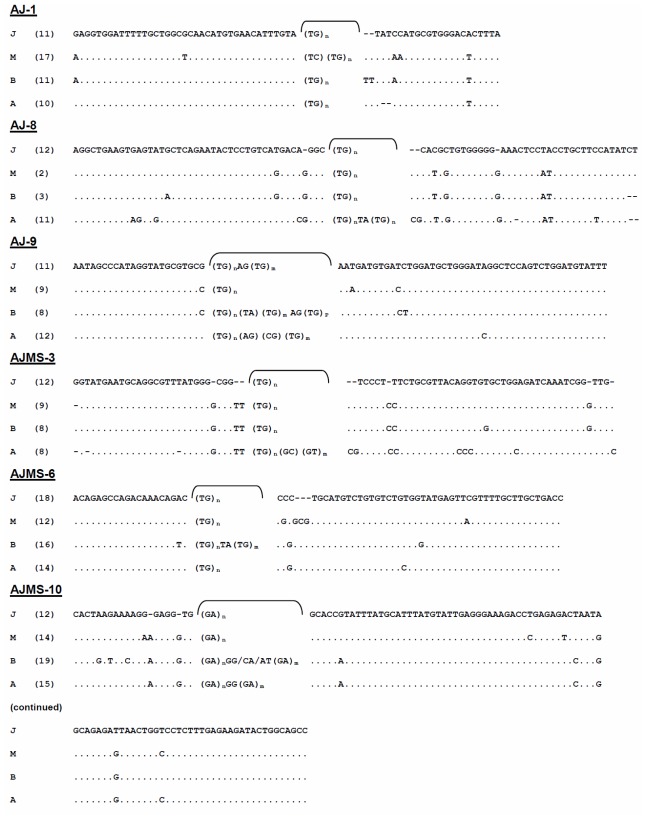
Alignment of consensus sequences of six microsatellite loci from four *Anguilla* species (J, *A. japonica*; M, *A. marmorata*; B, *A. bicolor pacifica*; and A, *A. anguilla*). Numbers of WebLogo sequences from homologous loci are shown in parentheses. The bracketed regions are major motif sequences. n, m and p are repeat numbers.

In each species, six consensus sequences were built up from all of the homologous sequences of the six loci, and they were then combined together. Lengths of the four consensus sequences ranged 458–469 bp. Mean A + T components (51.16%) were slightly higher than G + C ones (48.84%). In total, 68 point mutation sites were found among the four *Anguilla* eels with variations including 61.97% substitutions and 38.03% indels. All 27 indel sites, including 23 monomorphic and 4 polymorphic sites, and 44 substitution sites, were present at the consensus sequences of the six loci among the four *Anguilla* species. Some particular dinucleotide mutations were only present in certain species. For example, an insertion of CG was only observed at loci AJMS-3 (positions 31 and 32) and AJ-8 (positions 45 and 46) in *A. anguilla*, and at locus AJMS-6 (positions 25 and 26) in *A. marmorata* (Figure 3). Single-nucleotide substitutions were extensively distributed at the six microsatellite loci in the four species. A K substitution was found at position 20 of the AJ-1 locus in *A. marmorata*. More substitutions were found at alleles of the AJ-8 locus in *A. anguilla* than in other species. For example, a substitution of A/G was specifically observed at positions 11, 12, and 15, and C/A at position 40. A specific G/C substitution (position 23) at locus AJ-9 was only found in the two tropical species but not in the temperate eels. There were three specific substitutions at positions 5, 7, and 9 of the AJMS-10 locus in *A. bicolor pacifica*. In summary, the mutation patterns in the flanking regions primarily resulted from indels and substitutions. Indels in the flanking regions are believed to be one cause that results in allelic size variations.

**Figure 4:**
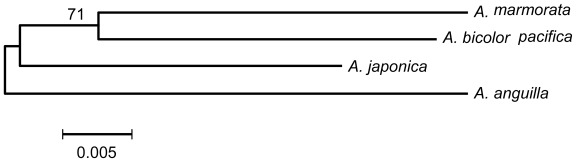
Phylogenetic tree constructed by the Neighbor-joining method. Bootstrap values were obtained by 1000 replicates.

Numbers of transitions (Ts) between species in the combined consensus sequence ranged from eight (*A. japonica vs. A. marmorata* and *A. marmorata vs. A. bicolor pacifica*) to 11 (*A. anguilla vs.* other species). Numbers of transversions (Tv) ranged from 12 (*A. japonica vs. A. anguilla* and *A. marmorata vs. A. bicolor pacifica*) to 16 (*A. anguilla vs. A. marmorata*). These results implied that fewer substitutions appeared in tropical *A. marmorata vs. A. bicolor pacifica* than in temperate *A. japonica vs. A. anguilla*. The interspecific genetic distances obtained from the K2P genetic model ranged from 0.044 (*A. marmorata vs. A. bicolor pacifica*) to 0.061 (*A. marmorata vs. A. anguilla*) (Table 2). The NJ topology among the four *Anguilla* eels indicated that *A. marmorata* had a closer evolutionary relationship with *A. bicolor pacifica* than with temperate eels, and *A. japonica* was not clustered with *A. anguilla* (Figure 4). These results implied that interspecific allopatric evolution existed between these two temperate eels.

### 2.3. Evolutionary Mechanism of Microsatellite Loci

Changes in repeat numbers of major regions, substitutions in entire sequences, and indels in flanking regions, are principle reasons resulting in polymorphisms within microsatellite loci. Previous reports indicated two mutation models that may cause changes in repeat numbers and lead to microsatellite instability. One model is unequal crossing-over that is the result of a recombination between homologous chromosomes that are misaligned. The alternative model is slip-strand mispairing errors that occur during DNA replication [31]. When major regions of perfect microsatellites produce a single-nucleotide substitution, it may result in interrupted microsatellites. In this study, several identical single-nucleotide substitutions were present in these *Anguilla* species (Table 1). For example, an interrupted major region, (TG)_n_ AG (TG)_m_, at locus AJ-9, was found in all of these *Anguilla* species except *A. marmorata*. In contrast, some specific, interrupted mutations were only discovered in particular species. For instance, a TA substitution in the major region of the AJMS-6 locus was specifically present in *A. bicolor pacifica*, but not in other species. As a result, this sequence character can be considered a good genetic marker to identify *A. bicolor pacifica*. However, these mutations have induced high genetic divergences within and among *Anguilla* species.

In this research, most homologous alleles of these microsatellites sequenced across species revealed that the major mutational event was a change in the repeat number, but these interrupted sequences from the same or different species of *Anguilla* also produced homologous allelic sizes. For example, the major regions at locus AJ-1 with TC (TG)_6_ and (TG)_7_ expressed an identical allelic size in *A. marmorata*. The sequence of the interrupted repeat at locus AJ-9, (TG)_8_ AG (TG)_10_, in *A. japonica* had the same sequence length as (TG)_19_ in *A. marmorata*. Most of the interrupted sequences were found in *A. bicolor pacifica*, *A. anguilla*, and *A. marmorata* rather than in *A. japonica* (Table 1), which suggests that greater complexities were present in those three species, in contrast to a rather simple form in *A. japonica*. The lack of a significant relationship existing between genetic distances and DNA complexity in these microsatellites suggests it is possible that substitutions randomly occurred within major regions of these microsatellites. In this study, indels were not found in major regions of the six microsatellites from these *Anguilla* eels. Consequently, random mutations are another factor which can cause variations in complexity among different species. Stephan reported that a single-nucleotide indel did not occur in any major region of microsatellite loci, which indicated that principal mutational patterns of microsatellites involved changes in the repetition number by the two cardinal mechanisms of slippage-strand mispairing and unequal crossing-over, and random substitutions within major regions [32]. Those inferences of evolutionary mechanisms are consistent with the results of this study. All these results imply that parallel evolution is present in these *Anguilla* eels. Kuittinen *et al.* described a parallel pattern of microsatellite sequence variations within and between populations of *Arabidopsis thaliana* [33]. A similar result was also revealed at the *Anguilla* species level (Figure 2, Table 1).

In fish, indels and substitutions are fairly frequent in flanking regions of microsatellite loci. Blankenship *et al*. observed point mutations and typed 668 different microsatellite flanking-sequence haplotypes from Chinook salmon [34]. In addition, microsatellite size variants due to indels in flanking regions were previously described [18,35]. In this study, indels were also found in flanking regions of five microsatellite loci (but not locus AJ-9) (Figure 3) and produced greater divergence of allelic sequences. Our results implied that mutational processes at these loci are probably far more complex than expected from the simple model of changes in the number of repeat units. There were no unique mutant patterns present in temperate or tropical freshwater eels.

### 2.4. Are *A. japonica* and *A. anguilla* a Sister Group?

Patterson estimated that *Anguilla* originated approximately 50–60 million years ago (Mya) during the Cretaceous-Eocene period, which is compatible with an Eocene *Anguilla* fossil collected from the Ypresian Stratum at Montevorca, Italy [36]. Based on the “Tethys Corridor hypothesis”, ancestral eels entered the Atlantic Ocean before the closure of the Tethys Sea (in the Oligocene, ca. 20~30 Mya) [37]. Using molecular dating, Aoyama and Tsukamoto determined that the two Atlantic *Anguilla* eels and *A. mossambica* diverged from a common ancestor at least 20~30 Mya [38,39]. However, we conjecture that the existence of these loci within *Anguilla* genomes must exceed 30 Mya, when the genus *Anguilla* may have first begun to diversify. In this study, six cross-specific microsatellite loci were successfully amplified from *Anguilla* eels. The conservation of a basic structure revealed by the sequence analysis among all species confirmed the homology of these loci within the genus *Anguilla* (Figure 3). Thus, the flanking sequences of the six microsatellite loci can also be used to clarify phylogenetic relationships of freshwater eels.

Ege subdivided *Anguilla* eels into four distinct groups, and the largest group with a long dorsal fin and uniform coloration included *A. rostrata*, *A. anguilla*, *A. japonica*, *A. mossambica*, *A. dieffenbachii*, and *A. borneensis* [3]. It was inferred that all six species within this group were more primitive than the others. They dispersed into different oceans before the closure of the Tethys Sea, with the subsequent formation of particular migration loops and speciation models once they were established in various oceans. In this study, we analyzed the phylogenetic relationship of four *Anguilla* eels using flanking sequences from six microsatellite loci. Although *A. anguilla* and *A. japonica* have similar adult-phase morphological characteristics and temperate habitats, our results suggested that *A. japonica* was not clustered with *A. anguilla* in the phylogenetic tree (Figure 4). The result is very similar to that inferred from mtDNA [10].

Most *Anguilla* species inhabit the Indo-Pacific Ocean, and it is firmly believed that this ocean is the center of their speciation. Marble eels were the next group to occupy the Indo-Pacific Ocean. *Anguilla marmorata* is considered to be the most primitive type among the marble eels, and it has the broadest species range which extends from southern Japan to southeastern Africa. However, if sympatric evolution seems logical, we can clearly imagine that *A. bicolor pacifica*, *A. marmorata*, and *A. japonica* have more-similar genetic components than those of the allopatric *A. anguilla*. These results seem to better conform to earlier results.

## 3. Experimental Section

### 3.1. Sampling

Ten specimens of *A. japonica* were caught in the estuary of the Tanshui River in northern Taiwan (N25°15′, E121°25′); 10 specimens of *A. anguilla* originally sourced from Europe were donated by a fish farm in northern Taiwan; and 10 specimens each of *A. bicolor pacifica* and *A. marmorata* were collected from Hueá, Vietnam (N16°45′, E107°30′).

### 3.2. Microsatellite Cloning and Sequencing

Muscle tissues of all individuals were immediately preserved in 95% ethanol after being caught until DNA extraction. Ethanol was removed from the tissues by evaporation before further treatment. The tissue (500 mg) was digested overnight in 1 mL lysis buffer (10 mM Tris-HCl (pH 8.0), 2 mM EDTA, and 10 mg/mL dithiothreitol) and 55 μL of proteinase K (0.5 mg/mL) at 55 °C before DNA extraction. DNA was extracted following standard procedures [40]. Genomic DNA was quantified and diluted to a working concentration of 1 ng/μL. We chose six conserved GA/GT dinucleotide microsatellite loci cloned from the *A. japonica* genome. The AJMS-3, -6, and -10, and AJ-1 and -8 microsatellite sequences in EMBL (with the respective accession numbers of AJ297601, AJ297603, AJ297605, AJ845112, and AJ845113) were described previously [41,42]. The AJ-9 microsatellite locus was freshly cloned from the *A. japonica* genome for this study, and its accession number is AJ844913.

Six microsatellite loci from the four Anguilla species were amplified via a polymerase chain reaction (PCR). The PCR consisted of approximately 5 ng genomic DNA, 50 pmol of a reverse primer, 50 pmol of a forward primer, 25 mM dNTP, 0.05~0.1 mM MgCl_2_, 10× buffer, and 5 U Taq polymerase (Takara, Tokyo, Japan), brought to a 125-μL volume with Milli-Q water. We ran one cycle of 4 min at 95 °C; 8 cycles of 30 s at 95 °C, 30 s at 50 °C, and 30 s at 72 °C; and 35 cycles of denaturing for 30 s at 95 °C, primer annealing for 30 s at 52~56 °C (the temperature varied depending on the primers and species), and a further extension of 30 s at 72 °C. We evaluated 10 μL of each product on a 2% agarose gel to check the PCR success and confirm the product sizes. In an initial survey of these four species of Anguilla eels, primers for these loci were annealed, and PCR products for 70%~90% of the samples were produced.

The remaining PCR-amplified products were run on 1% agarose gels. Bands visualized using ethidium bromide were purified from the gel. Subcloning was used to isolate haplotypes for sequencing purposes. Purified DNA was cloned into a pGEM-T easy vector (Promega, Madison, WI, USA) for each subclone, five colonies on each plate were randomly selected, and plasmid DNA was isolated using a mini plasmid kit (Geneaid, Taichung, Taiwan). In total, 274 different sequences were determined on an Applied Biosystems (ABI, Foster City, CA, USA) automated DNA sequencer 377 (vers. 3.3) using a Bigdye sequencing kit (Perkin-Elmer, Wellesley, MA, USA). A T7 or SP6 primer was used in the sequencing reaction each time. PCR cycle parameters for sequencing were 35 cycles of 30 s at 95 °C, 30 s at 50 °C, and 60 s at 72 °C.

### 3.3. Data Analysis

Sequences of the six microsatellite loci from four *Anguilla* eels were aligned using the BioEdit software program [43]. Inter- and intraspecific variations in sequences were determined using DNASP software [44]. Mutant patterns at major regions of the six microsatellites from the four species were separately visualized with the naked eye. Microsatellites were divided into three categories of perfect, interrupted, and compound types, based on the composition of their major regions [19]. All flanking regions of the microsatellite sequences were freshly aligned and analyzed. The consensus sequences were generated by combining information from point mutations of various intraspecific flanking regions at each locus with the WebLogo program [45]. All consensus sequences from homologous loci were combined and aligned, and interspecific variations were analyzed with the BioEdit [43] and MEGA programs [46]. Numbers of transitions (Ts) and transversions (Tv) were calculated with DAMBE software [47]. Genetic distances were computed, based on the Kimura two-parameter (K2P) model [48]. The topology was constructed using the Neighbor-joining (NJ) [49] method and bootstrap values were obtained by 1000 replicates [50].

## 4. Conclusions

Mutational patterns reported within the flanking and major regions of six microsatellite loci in these *Anguilla* eels demonstrated that slipped-strand mispairings, substitutions, and random point mutations were the major mechanisms creating microsatellite diversity. No specific mutation occurred in tropical or temperate freshwater eels; that is to say, all patterns implied interspecific parallel evolution of microsatellite sequences present among these *Anguilla* species. According to the NJ topological analysis of microsatellite sequences, the hypothesis of *A. japonica* and *A. anguilla* being a sister group must undeniably be rejected.

## Figures and Tables

**Table 1:** Annealing temperatures (*T*_a_) used in the PCR amplification and diversity of the repeat regions from six microsatellite loci of four *Anguilla* freshwater eels.

Locus *Species*	*T*_a_	No. of Sequences	Repeat Regions
**AJ-1**			
*A. japonica*	58	11	(TG)_7~13_
*A. marmorata*	54	17	(TG)_6, 7, 8, 10_; TC(TG)_6_
*A. anguilla*	54	10	(TG)_5~10_
*A. bicolor pacifica*	54	11	(TG)_7~9_
**AJ-8**			
*A. japonica*	56	12	(TG)_14~18, 20_
*A. marmorata*	52	2	(TG)_14, 15_
*A. anguilla*	52	11	(TG)_12, 13, 15, 17, 19_; (TG)_6_(TA)(TG)_11_
*A. bicolor pacifica*	52	3	(TG)_13, 22_
**AJ-9**			
*A. japonica*	54	11	(TG)_8, 9, 11, 12, 14, 15_; (TG)_8_AG(TG)_10_
*A. marmorata*	56	9	(TG)_14, 15, 17, 18, 19, 23, 27_
*A. anguilla*	54	12	(TG)_12_; (TG)_10, 19, 21_AG(TG)_4_
			(TG)_3_CG(GT)_10,11_
			(TG)_7_CG(TG)_4_AG(TG)_6_
*A. bicolor pacifica*	54	8	(TG)_9, 10, 13, 14_(AG)_1, 2_(TG)_9~12_
			(TG)_5_TA(TG)_3_AG(TG)_11_
**AJMS-3**			
*A. japonica*	56	12	(TG)_7~10_
*A. marmorata*	56	9	(TG)_7, 9, 10_
*A. anguilla*	54	8	(TG)_7, 10, 11_; (TG)_3, 8_CG(TG)_1, 4_
*A. bicolor pacifica*	54	8	(TG)_7, 8, 10_
**AJMS-6**			
*A. japonica*	56	18	(TG)_9, 12~16, 18_
*A. marmorata*	54	12	(TG)_12, 13, 14, 16, 18, 19_
*A. anguilla*	54	14	(TG)_7, 10, 13, 14, 15_
*A. bicolor pacifica*	54	16	(TG)_12~18_TA(TG)_0, 2_
**AJMS-10**			
*A. japonica*	58	12	(GA)_10, 12, 14~16, 18~22, 35, 36, 39_
*A. marmorata*	54	14	(GA)_8~12, 14, 15, 17_
*A. anguilla*	54	15	(GA)_22_
			(GA)_9, 12, 13, 15, 16, 17_GG(GA)_6, 9, 10, 11,12_
*A. bicolor pacifica*	54	19	(GA)_13~15,17,19, 20, 22, 23, 26_
			(GA)_6, 7_GG(GA)_5, 12_
			(GA)_7_CA(GA)_12, 13_; (GA)_9_AT(GA)_9_

**Table 2:** Genetic distances and standard errors (above the diagonal) and number of differences (below the diagonal) among consensus sequences of four *Anguilla* eels.

	*A. japonica*	*A. marmorata*	*A. bicolor pacifica*	*A. anguilla*
*A. japonica*	---	0.050 ± 0.11	0.055 ± 0.011	0.053 ± 0.011
*A. marmorata*	22	---	0.044 ± 0.010	0.061 ± 0.012
*A. bicolor pacifica*	24	20	---	0.057 ± 0.011
*A. anguilla*	23	27	25	---
